# Strain in a silicon-on-insulator nanostructure revealed by 3D x-ray Bragg ptychography

**DOI:** 10.1038/srep09827

**Published:** 2015-05-18

**Authors:** V. Chamard, M. Allain, P. Godard, A. Talneau, G. Patriarche, M. Burghammer

**Affiliations:** 1 Aix-Marseille Université, CNRS, Centrale Marseille, Institut Fresnel UMR7249, 13013 Marseille, France; 2 Laboratoire de Photonique et de Nanostructures, CNRS, 91460 Marcoussis, France; 3 European Synchrotron Radiation Facility, BP220, 38043 Grenoble, France; 4 Institut P' (UPR 3346 CNRS), Université de Poitiers, ENSMA, Bd Pierre et Marie Curie, 86962 Futuroscope, France; 5 Department of Analytical Chemistry, Ghent University, Krijgslaan 281, S12, 9000 Ghent, 9000 Ghent

## Abstract

Progresses in the design of well-defined electronic band structure and dedicated functionalities rely on the high control of complex architectural device nano-scaled structures. This includes the challenging accurate description of strain fields in crystalline structures, which requires non invasive and three-dimensional (3D) imaging methods. Here, we demonstrate in details how x-ray Bragg ptychography can be used to quantify in 3D a displacement field in a lithographically patterned silicon-on-insulator structure. The image of the crystalline properties, which results from the phase retrieval of a coherent intensity data set, is obtained from a well-controlled optimized process, for which all steps are detailed. These results confirm the promising perspectives of 3D Bragg ptychography for the investigation of complex nano-structured crystals in material science.

Strain fields in semiconductors are long proposed as a route for the design of well-defined electronic properties in microelectronic devices^1^. It relies on the modification of the electronic band structure, reported for instance in silicon^2^ or in germanium^3^,^4^. The case of the integration of strained semiconductors to the widely used silicon-on-insulator (SOI) technology is of particular importance as the SOI technology offers the production of high-quality low-cost substrates associated to gains in device design, electronic performance and scalability^5^. In this context, the precise control of strain is mandatory. It includes the detailed knowledge of the crystal lattice behavior within a given nanostructure morphology *and* with regards to the fabrication process. Indeed, not only the presence of surfaces and interfaces is susceptible to dramatically alter the expected electronic design, but the nanostructure elaboration itself^6^. This calls for experimental methods able to image the crystalline properties in three dimensions (3D), over a large field of view, within a nanometer resolution and -ideally- in a non-pertubative way. Experimental approaches such as transmission electron microscopy^7^, x-ray Bragg nano-diffraction^8^and finite-support Bragg coherent diffraction imaging^9^are well-established techniques for strain analysis. They however only partly fulfill the required specifications justifying the development of an imaging approach capable of entirely answering this need.

The microscopy technique that we promote, namely Bragg ptychography, is an extension of the ptychography method which was originally implemented for electron microscopy applications^10^. It makes use of multiple exposures that combine redundant information from partially overlapping illumination areas onto an extended sample^11^. A set of partially redundant far-field x-ray coherent intensity patterns is recorded while the sample is translated through the finite-sized beam spot. As the measurement gives access only to the intensity (*i.e.* the squared amplitude) of the scattered wave-field, the image reconstruction relies on numerical approaches using iterative algorithms^12^. The retrieved complex-valued image is hence quantitative and contains information directly related to structural parameters^13^. The main advantage of ptychography with regards to other x-ray lens-less microscopy approaches^9^,^14^ is its compatibility with the imaging of samples much larger than the beam^15^,^16^. Indeed, x-ray beam sizes are primarily limited by the coherent lengths associated to the x-ray source and/or detection scheme. They can be further decreased with focusing optics when the optimization of the flux at the sample position is required for the analysis of weakly scattering specimen. In addition, as ptychography is based on the deconvolution of the illumination and the sample scattering contrast functions, gains in resolution and sensitivity are achieved with regards to the ones obtained with the same experimental set-up used in a scanning mode approach^17^,^18^. Finally, in the particular case of the Bragg geometry, *i.e.* when the measurement is performed in the vicinity of a crystal Bragg reflection, ptychography acquires intrinsic 3D capabilities^20^,^21^,^22^ together with, in principle, high sensitivity to 3D crystalline displacement fields.

The possibility to image 3D extended crystals in a non invasive way using x-ray Bragg ptychography was proposed^19^ and recently experimentally demonstrated^20^,^21^,^22^. However, the capability to access a relevant and therefore *exploitable* description of the 3D crystalline properties (*e.g.* displacement fields) at the nanoscale is still lacking in the Refs. [20–22]. In 2D, Hruszkewycz *et al.* demonstrated the possibility to extract high fidelity structural information using Bragg projection ptychography^23^,^24^. Here, we show that a highly controlled experimental procedure allows to gain the ultimate robustness and sensitivity needed to produce a 3D high-fidelity image of the crystalline properties. This ultimate control is achieved through an accurate knowledge of the x-ray illumination beam together with an optimized inversion procedure tested on shot-noise corrupted numerical data-sets. This crystal microscopy, applied to a lithographically patterned SOI structure, reveals displacement fields with details hardly accessible by other means.

## Results

### Experiments

**Figure 1:**
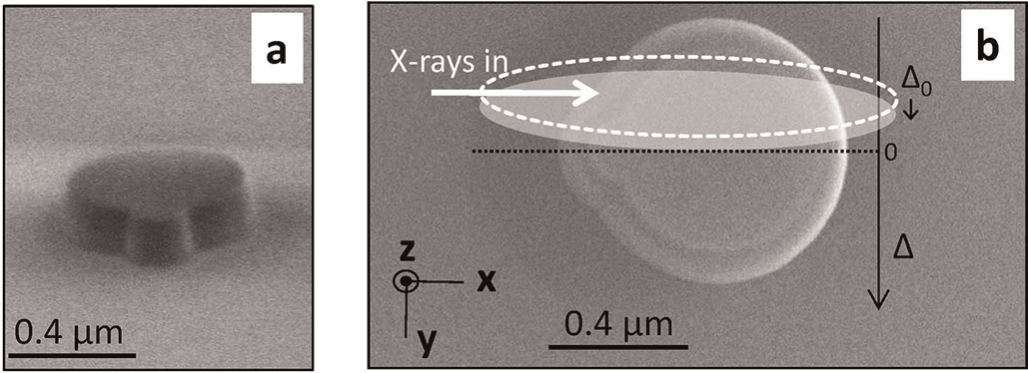
The crystalline silicon-on-insulator (SOI) structure as seen by scanning electron microscope. (a) Front view. (b) Same as (a), top view; The direction of the ptychography translation 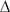 (black arrow) and the scanning step 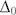 are indicated. The projected direction of propagation of the incident beam (white arrow) together with the beam footprint (FWHM of intensity, dotted ellipse) are shown. The gray ellipse corresponds to the beam footprint at the next beam-to-sample position. The (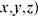 orthogonal laboratory frame is given.

The crystalline structure used for this study is patterned from a SOI wafer, whose top crystal, a 0.18*μ*m thick layer, is 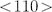 oriented (see Methods). Scanning electron microscopy views of the structure external shape are presented in Figure 1. The shape of the patterned structure corresponds to the juxtaposition of two disks, a small and a large one, both of them presenting inclined edges. Their diameters at the Si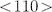/SiO_2_ interface are 0.77*μ*m and 0.26*μ*m, respectively. Although very simple, this shape exhibits only one symmetry plane, whose normal will be used as the translation direction during the ptychography experiments.

**Figure 2:**
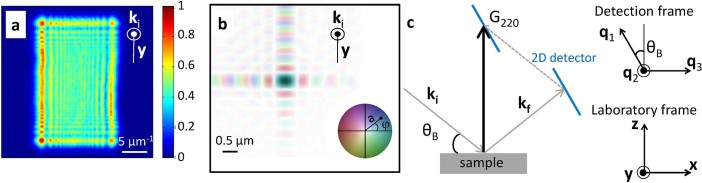
Experimental considerations for 3D Bragg ptychography: beam profile and Bragg diffraction geometry. (a) Intensity pattern of the over-focused direct beam (arbitrary units) measured with a high-resolution camera. (b) Color rendition of the complex-valued beam profile, retrieved from the inversion of (a) and shown in the Fresnel zone plate focal plane. The brightness and color correspond to the linear scale amplitude 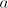 and to the phase 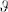, respectively. (c) Description of the 3D Bragg diffraction geometry, including the Bragg angle (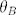), the incident and exit wave vectors (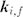, respectively) and the Bragg vector (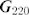). The 3D non-orthogonal (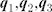) detection frame is defined in agreement with the detection acquisition modality, corresponding to the 2D detector plane and to the rocking curve direction. The (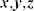) laboratory frame is also shown.

One major parameter of a ptychography experiment is the beam illumination function, whose knowledge is used for the deconvolution operation performed during the ptychography inversion. Although blind deconvolution has been demonstrated for the analysis of 2D ptychography intensity patterns^25^,^26^, we observed, for 3D Bragg ptychography, a significant increase of the retrieved image quality when the illumination is known^21^. This can be intuitively understood by comparing the dimensionality of the problem to solve (the 3D phase of each intensity pattern) to the one of the acquired redundancy, which is only 2D at most (the two possible directions of the beam-to-sample scanning translations). Hence, prior to the 3D x-ray Bragg ptychography experiment itself, a detailed characterization of the profile of the x-ray nano-beam delivered by the synchrotron experimental set-up was performed. The coherent nano-beam was produced by a partially and asymmetrically illuminated focusing lens^27^. In order to image the complex-valued illumination function at the focal plane, a lens-less microscopy method was used, based on the simple and fast measurement of the over-focused direct beam intensity pattern, *i. e.*, the freely propagated beam far from the focus. This latter was further phased with an error-reduction algorithm^28^, wherein the wave propagation was performed by the Fresnel propagator. This approach requires the use of a well-defined support in a plane conjugated to the detection one (termed the numerical window), in order to introduce a significant amount of known information (*i.e.*, zero padding) allowing for the algorithm to converge. The ratio between the sizes of the numerical window and the support directly increases with the sampling frequency of the diffraction pattern. In our experimental set-up, this well-defined support constraint was given by the slits defining the lens aperture. Hence, to guarantee that the numerical window located at the slit plane was much larger than the slit aperture (*i.e.*, that the intensity was oversampled), a high-resolution camera, with pixel size typically in the micrometer range, was used (see Methods). Figure 2a shows the over-focused direct beam intensity pattern, where the observation of the high frequency oscillations resulting from the slit truncated beam, demonstrates that the data quality fulfills this oversampling criterion. The resulting retrieved illumination function is shown in Figure 2b (see Methods). The asymmetry in the slit aperture results in an elongated central spot, with size of 0.75 × 0.56*μ*m^2^ (distance between the two first zeros), along the horizontal and vertical directions, respectively, while the full-width-at-half-maximum (FWHM) of the central spot intensity is 0.31 × 0.23*μ*m^2^. Beside providing valuable information for our Bragg ptychography experiment, we want to underline the convenience of this nano-beam characterization method, similar to the one presented in Refs. 29,30, which is fast and robust as long as the data oversampling is verified. It permits a high flexibility in the characterization of the beam profile during long-lasting synchrotron experiments.

**Figure 3:**
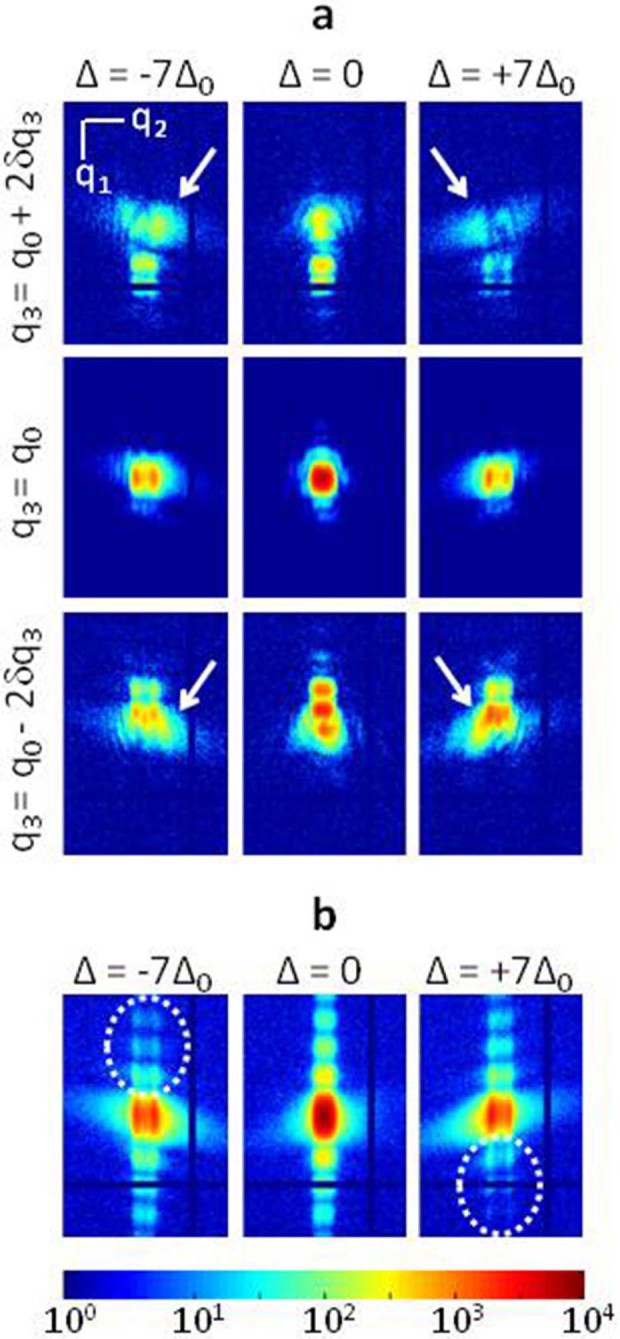
3D Bragg ptychography data set. (a) Intensity patterns extracted from the 4D coherent Bragg diffraction measurements. For each frame, measured in the 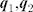 plane, only the central part of the pattern is shown. The steps along 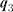 correspond to 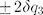, relatively to the most central position 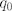 while along the ptychography translation 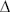, the steps are 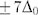 (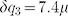
m^−1^ and 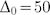 nm). The intensity values in 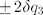 have been increased by a factor of 4 for sake of clarity. The white arrows emphasize the stronger intensity lobes, which are arising from the structure edges. (b) Intensity integrated along the 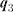 direction, for a fixed 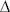, identical to (a). The dotted ellipses emphasize the missing intensity along the vertical streaks. In (a) and (b), the vertical and horizontal zero intensity lines correspond to blind pixels in the detector. The common logarithmic photon scale is shown in (b).

The 3D Bragg diffraction ptychography data set was then acquired in the vicinity of the silicon 220 Bragg reflection (noted ***G***_220_, with 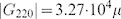
m^−1^), following the detection scheme shown in Figure 2c. Details about the acquisition procedure can be found in the Methods section. Due to the incidence angle 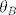, the beam footprint was elongated (1.2 × 0.23*μ*m^2^, Figure 1b) so that the structure was fully illuminated along the scattering plane, *i. e* the plane defined by the incident and exit wave vectors (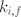, respectively). With this specific geometry, the whole structure could be investigated by scanning the beam-to-sample position 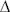 along the axis normal to the scattering plane. The scanning step 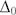 was fixed to 50 nm, ensuring enough overlapping between two successive positions. Typical coherent diffraction patterns acquired at different 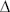 values and plotted in the detection frame, that is (**q**_1_,**q**_2_,**q**_3_) as shown in Figure 2c and described in Methods, are presented in Figure 3. Note that the most central position along q_3_ is slightly shifted by 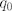 with regards to the origin of the reciprocal space. The continuity and reproducibility of the different features observed in the diffraction patterns, further emphasized in the integrated intensity patterns of Figure 3b, are strong evidences of the absence of beam drift or radiation damage.

### Analysis of the 3D data set

Before we present the result of the ptychographic reconstruction, we show in the following the detailed analysis of the intensity data set. In addition to allowing for the estimation of the data quality, the detailed observation of the intensity data set brings valuable information on the sample crystalline properties. In particular the presence of a displacement field in a crystal is directly revealed by its Bragg diffraction pattern. This relies on a simple property of the Fourier transform, which is well adapted to describe the propagation of the scattered wave from the sample plane to the far-field detector. Fourier transforming a purely real function leads to a centro-symmetric intensity pattern. This situation corresponds to the strain-free crystal case^9^. On the contrary, when an arbitrary displacement field is present in the crystal, it can be modeled by a phase field in the sample (complex-valued) scattering contrast^31^. This imaginary component breaks the centro-symmetry of the diffraction pattern, with regards to 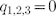
9.

This kind of behavior is observed in our data set. It is becoming more obvious when the beam is moving away from the central position, that is when one of the edges of the structure is strongly illuminated. This is shown in Figure 3a for 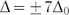. For 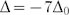, the strongest intensity lobe, emphasized by the white arrows and arising from the presence of the inclined edges, is observed for 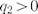, for all 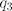 values. This corresponds to the situation where the centro-symetry of the 3D intensity pattern is broken with regards to the center of the Bragg peak (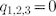). A similar situation is observed for 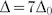. However, the fact that the intense lobe is observed for 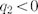 (and not 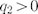) is the signature of a phase field of opposite sign with regards to the 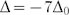 case. On the contrary, when the central part of the structure is illuminated (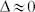), the most intense lobe is appearing on opposite sides of the diffraction pattern for 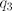 increasing from negative to positive, a situation closer to the strain-free case. This whole behavior (centro-symmetry for the central illumination position and break of centro-symmetry with opposite behaviors at the edge illumination positions) argues in favor of the presence of a displacement field at the edge of the silicon structure. Finally, possible refractions effects have been estimated and found to be negligible in the SOI structure^32^.

A second specific behavior is evidenced on the plots of the intensity patterns that were obtained after integration along 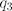 at each fixed 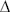. They are shown in Figure 3b for the same 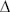
s as previously selected. At 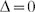, the vertical streak along 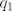, corresponding to the thickness interference fringes, presents a symmetric distribution with regards to 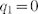. This symmetry is broken at other beam to sample position. In 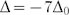, the streak is weaker for 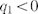 (dotted ellipse in Figure 3b). This behavior is specific to the presence of a displacement field along the sample thickness. The fact that the opposite situation is observed for 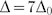 shows that this displacement is not constant along the translation direction.

**Figure 4:**
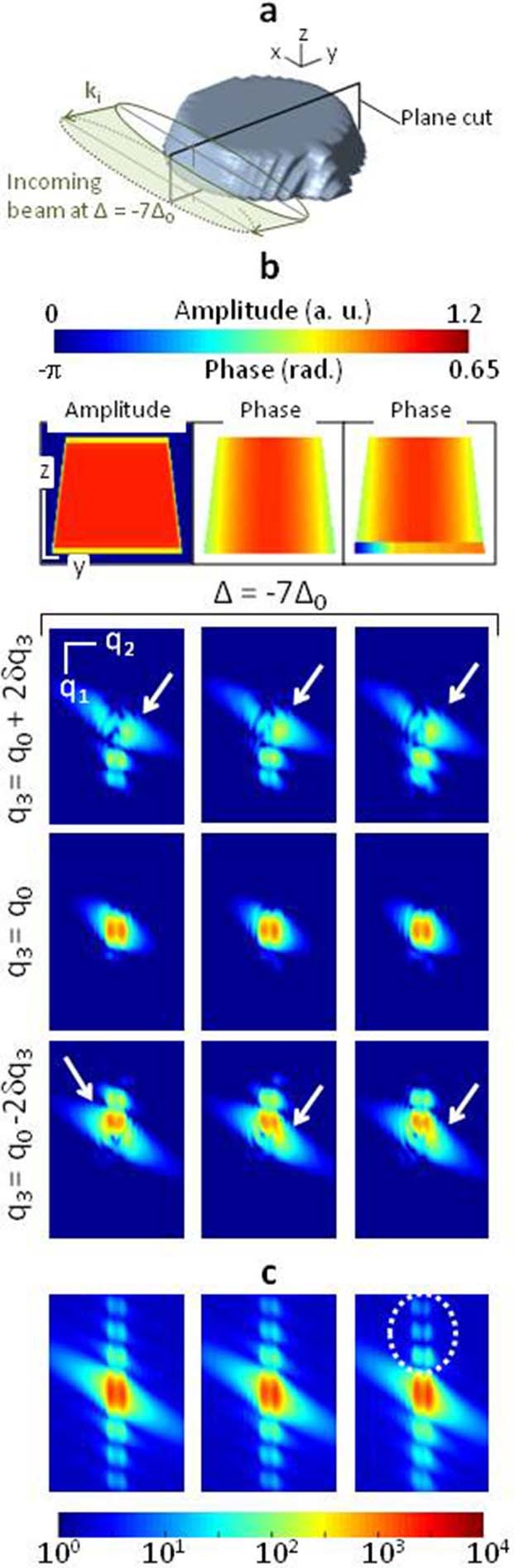
Presence of a crystalline displacement field: numerical studies. Estimation of the expected diffraction patterns calculated for different 3D strained crystals with shape similar to the SOI structure. (a) Common 3D iso-surface rendering of the synthetic object together with the incoming beam shape (FWHM of intensity) for 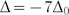. The laboratory frame is given; the length of the black lines is 100 nm. (b) Three synthetic models, corresponding to three different strain states and their corresponding diffraction patterns. The 2D sample description is shown in the plane indicated in (a) while the diffraction patterns are taken at the same 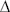 and 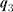 values as the ones of (Figure 2, left column). (c) Intensity integrated along the 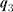 direction, for the same 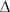 value. The specific features of the calculated diffraction patterns are emphasized by the white arrows and the dotted ellipse. The three strain states are as followed: (*Left*) The 3D strain-free crystal case. A 2D cut through the 3D amplitude is shown in (a). Note the assymetry in the spatial scale, which is underlined by the white lines, representing a 100 nm length. (*Middle*) Same calculation, obtained for a strained crystal: a displacement field with a radial symmetry is introduced at the edge of the structure. A 2D cut through the corresponding sample phase is shown at the top. (*Right*) Same as before with the simultaneous introduction of the displacement field at the edges and at the interface. This last model produces diffraction patterns in good agreement with the experimental ones.

Numerical simulations were performed in addition to the previous data analysis in order to evaluate qualitatively which displacement is producing a visible signature in the intensity data-set. This is particularly relevant when dealing with limited signal to noise ratio intensities. In the following, the expected Bragg intensities calculated for different empirical crystalline strain states and using numerical parameters as close as possible to the experimental conditions (sample shape and size, illumination function, sampling) are analyzed. The diffraction patterns shown for 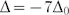 and plotted at reciprocal space coordinates identical to the ones used in Figure 3 are presented in Figure 4, for an intensity dynamical range comparable to the experimental data one. Three sample models have been chosen, with the same 3D shape and density, shown in Figure 4a. In Fig 4b and c, the strain-free case is first presented on the *left* column. The average intensity distribution reproduces fairly the experimental data. One notes that the edge streak is more inclined than the experimental one, showing a slight discrepancy between the edge inclinations in the model and in the silicon structure. More interestingly, the more intense lobe is appearing on opposite sides of the diffraction pattern when 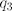 increases from negative to positive, as expected for a centro-symmetric diffraction pattern produced by a strain-free crystal. The vertical streak evidenced in the integrated intensity pattern of Figure 4c is equally distributed along **q**_1_. The *middle* column corresponds to the introduction of a displacement field developing at the edges of the structure. This leads to a break of the centro-symmetry, resulting in intensity lobes behaving similarly to the ones experimentally observed. However, the vertical streak visible in the integrated intensity plot of Figure 4c is still symmetric. The final introduction of an additional displacement at the interface (*right* column) breaks this symmetry along the thickness streak, leading to the definitive modification of the diffraction pattern, in good qualitative agreement with the experiment. It demonstrates that these simultaneous displacement fields at the edges *and* at the interface of the structure result to clear and observable signatures in the experimentally accessible data set. Reciprocally, the experimentally observed diffraction patterns could be explained by these displacement fields.

This preliminary experimental data analysis finally allows to provide a relevant structural model, which is most likely representative of the structural features present in the SOI sample. Therefore, the optimization of the inversion procedure, which is presented in the following, is based on numerical tests performed on this very same structural model which is now the object to retrieve.

### 3D phase retrieval

**Figure 5:**
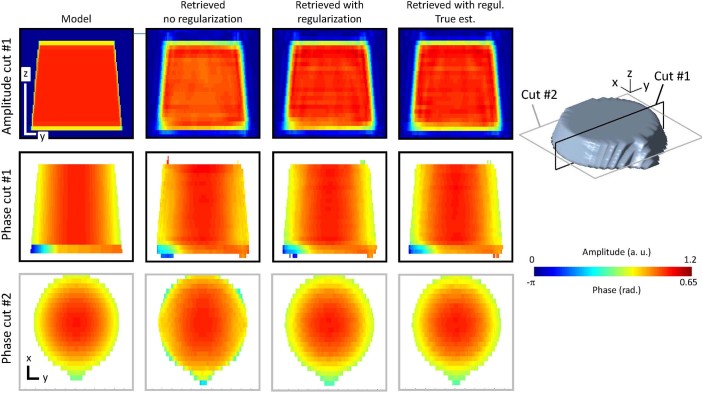
Optimizing the inversion scheme. (*Left*) The 3D synthetic model object used to test the inversion procedure. It corresponds to the model shown on the right column of Figure 4. (*Second column*) Retrieved image using a conjugate gradient optimization of the *Bouman and Sauer* maximum likelihood, initialized with the shape of the object. (*Third column*) Same as before introducing an additional regularization term to constrain the sample support. (*Right*) Same as before, initialized with the true synthetic object. The top, middle and bottom rows are different cuts of the 3D object, as defined on the 3D isosurface plot rendition on the right. The assymetric spatial scale is given on left (*y,z*) and (*x,y*) cuts. Each line corresponds to a 100 nm length. The sample density and the displacement field color scales are indicated on the right. The excellent agreement observed between the two last retrieved solutions shows that the found inversion process is optimum.

Before the inversion of the experimental data set is performed, the inversion procedure needs to be optimized and quantified to ensure the pertinence of the retrieved image and its physical interpretation. Indeed, any inversion process brings its own artifacts due to the presence of photon shot noise^33^. Understanding these effects requires a detailed analysis which allows to ensure that the best solution is found and permits to avoid over-interpretation of the retrieved image. Using the conclusion of the numerical simulation, different inversion procedures have been tested on an intensity data set produced by the third sample model of Figure 4, further described in Figure 5 (left column). Before inversion, the complete set of diffraction intensity patterns, calculated in the detection frame, was further corrupted by Poisson shot noise in order to take into account the limited amount of photons in the experiment.

The inversion of the 4D intensity data was performed by optimizing the approximation of the Poissonian likelihood proposed by *Bouman and Sauer* in Ref. [34] (see also Ref. [33] for details). This was done with a conjugate gradient algorithm chosen for its fast convergence property. The initial estimate was given by the shape of the synthetic model object (the phase was set to zero). Although the reconstruction shown in Figure 5 (second column) allows to recognize the sample structure, the image is clearly degraded, particularly with respect to the phase exact value in the vicinity of the edges. The obtained solution, which is of poor quality only, is therefore not acceptable for our purpose and calls for an improved inversion scheme. The next inversion corresponds to the same inversion process where an additional regularization has been introduced in order to bring reasonable physical information about the sample^21^. Here we choose to consider the thickness of the sample: the solution we are seeking at is contained only into a film-like support. Due to the limited number of photons resulting in an expected spreading of the object induced by resolution effects, the thickness of the film has to be slightly larger (10 %) than the true object thickness. As can be seen in Figure 5 (third column), this process increases clearly the quality of the reconstruction, which can be hardly distinguished from the synthetic model object, validating the capability of Bragg ptychography to retrieve with a *high fidelity* the displacement field. A final test is performed to further quantify the solution quality. The same inversion process is used with an initialization given by the synthetic model object. Indeed in this case, the only remaining discrepancies between the solution and the model are expected to result from the presence of shot noise in the data set: shot noise corrupted data are naturally producing a band-limited image of the sample and this latter is inevitably corrupted by the inversion procedure itself and its capability to deal with shot noise (*i.e.* the noise model). As can be seen on the right column of Figure 5, the retrieved solution in this last test is in excellent agreement with the solution obtained in the previous test. It confirms that the inversion process is optimum. Moreover, it gives a upper limit of the image quality expected for the experimental data. Based on this result, we used this last inversion process to phase back the experimental data set, without assumption on the strain state of the sample.

**Figure 6:**
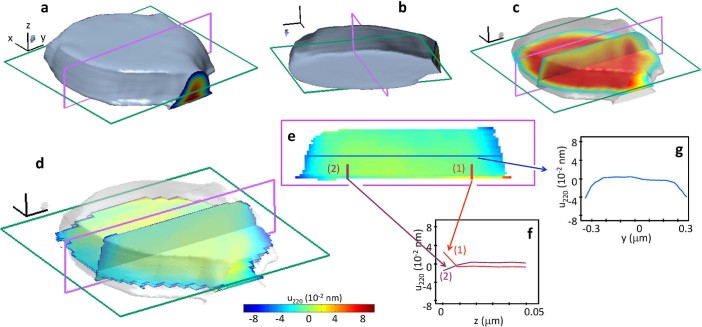
3D x-ray Bragg ptychography image of the SOI structure. (a) 3D Isosurface plot rendition of the retrieved crystalline SOI structure density, shown in the laboratory frame (threshold at 30%). The length of the frame black lines corresponds to 0.1 *μ*m. (b) Same as (a), other view. (c) Orthogonal 2D cuts of the density. (d) Orthogonal 2D cuts of the displacement field component 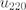. The color scale used to plot the 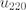 images is given at the bottom. (e) 2D cut in the (***y,z***) plane extracted from (d). The specific behavior of 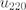 is emphasized in the 1D cuts taken along the colored lines in (f) and (g).

The whole set of experimental 3D intensity patterns is now analyzed with the optimized 3D Bragg ptychography algorithm (see Methods). The initial guess is the strain-free model object with external shape close to the SOI pattern nominal shape. The result of the reconstruction, plotted in the orthogonal laboratory frame is shown in Figure 6. As can be seen, the external shape is retrieved with a good agreement with the nominal object shape depicted by scanning electron microscopy (Figure 6a,b and Figure 1). The internal density is rather homogeneous (Figure 6c). Some internal fluctuations remain, related most likely to experimental imperfections (*e.g.*, positioning accuracy). Note as well the presence of a bit of aliasing in the data set, which results in a slight truncation of the object along its longer dimension. This could have been easily overcome with the approach developed in Ref. [21] and does not represent an intrinsic limit of the method. The 3D plot allows finally to quantify the resolution, using the presence of sharp edges along the three direct space directions. The resolution voxel size is estimated to about 50 × 45 × 15 nm^3^ along the x, y and z directions, respectively.

More interestingly, 3D Bragg ptychography gives access to the phase 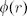 of the retrieved complex-valued quantity. This quantity holds in principle information about the crystalline properties of the lithographic structure and more precisely about the displacement field 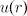 since^31^

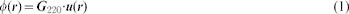
where 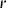 is the direct space coordinate. Hence, the projection of the displacement 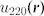 can be extracted from the phase map using 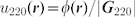. This is shown in Figures 6d and 6e. As expected from the raw data analysis and from the numerical simulations introducing strain, the measured displacement field exhibits visible variations at the edges of the structure and at the interface (emphasized in Figure 6f and 6g). This definitively confirms the capability of x-ray Bragg ptychography experiments to provide high fidelity images of displacement fields in 3D.

## Discussion

The aim of this work was to demonstrate that 3D Bragg ptychography is a microscopy technique able at imaging with strong accuracy the crystalline properties of nano-structured crystal and is able thereby to bring valuable information for the understanding of structural properties of complex crystalline nano-architecture. We think that our careful analysis, which includes the detailed investigation of the raw data, the strained crystal numerical simulations and the optimization of the inversion process, brings the decisive arguments to this demonstration.

**Figure 7:**
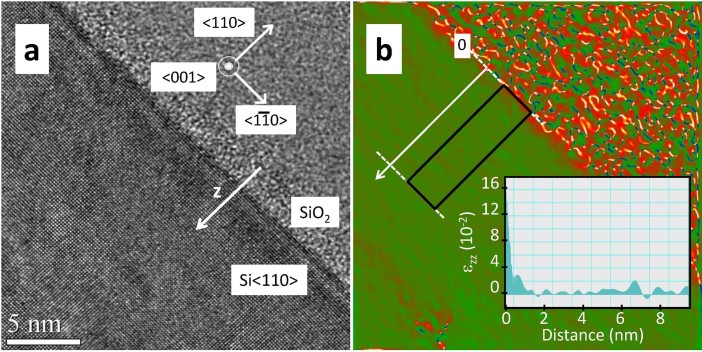
Transmission electron microscopy at the Si/SiO_2_ interface (a) Transmission electron microscopy image of the Si <110>/SiO_2_ interface measured on the un-patterned SOI wafer. The crystallographic directions of the Si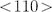 layer are indicated. (b) The 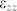 strain component extracted from (a), in absolute units. The inset shows the mean value of 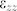 as a function of the distance to the interface, calculated in the region delimited by the black rectangle. An increase of 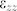 is observed near the interface.

Exploring the origin of the observed displacement field is out of reach of this work. A deeper understanding would require additional local structural information. Transmission electron microscopy (TEM) could provide the desired strain sensitivity and resolution, but the sample preparation, which requires the thinning down of the isolated SOI structure is particularly tricky and subjected to the introduction of relaxations or defects. The investigation of a series of patterned Si structure prepared from varying lithographic parameters is mandatory to explore the precise relationship between the crystalline structure and the sample shape, size and preparation processes. In spite of these difficulties, we can however argue that the crystalline behavior at the interface is likely related to the fabrication process of the SOI wafer. A similar interfacial structure was observed in Ref. [20]. In addition, the investigation of the considered interface, performed with TEM on the un-patterned SOI wafer, shows as well a strain field (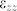) at the interface (Figure 7). However, the detailed comparison between the TEM and the x-ray results is vain due to the strong difference in the image resolution. Indeed, this requires the introduction of the resolution function parameters (resolution function model, relative position of interface in TEM and x-rays) for which a good agreement between TEM and x-rays images can always be found. In addition, finite element models (FEM) were performed in order to bring some insights to the presence of the displacement field located at the edge of the structure. This calculation is based on continuum elasticity theory, using the intrinsic properties of the materials (*i.e.* the elastic constants) and does not take into account the preparation history. The fact that the displacement fields could not be reproduced by FEM is an argument in favor of a lithographically induced strain field at the edges.

To conclude, we have shown that an optimized 3D x-ray Bragg ptychography method, including illumination function knowledge and regularized inversion procedure, possesses the capability to provide high fidelity images of strain fields in nanostructured crystals without dedicated sample preparation. These specificities are required for addressing pending problems in a wide variety of physical or biological material science. We expect that this method will generalize together with the development of the forthcoming x-ray sources and instruments.

## Methods

### Sample preparation

The SOI wafer is composed of a Si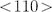 top layer (0.18*μ*m) and SiO_2_ layer (0.02*μ*m) on a Si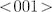 substrate. The patterning method involved e-beam lithography and SF_6_-based reactive ion etching to produce inclined edges. Due to the crystallographic orientation difference between the top layer and the substrate, it was possible to image solely the Si top structure.

### Experiment: beam profile determination

The x-ray experiment was performed at the ID13 beamline at ESRF (European Synchrotron Radiation Source) with a monochromatic beam of wavelength 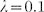 nm and bandwidth 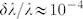. The finite-sized beam spot needed for the ptychography scan resulted from the focalisation of a fully coherent beam using a Fresnel zone plate (FZP) with focal length of 0.14m. For this purpose, the FZP aperture was reduced to 60 × 40 *μ*m^2^ in the vertical and horizontal direction, respectively, so that the illumination area matched the beam transverse coherence lengths. As the FZP central part was occluded by a beam stop to avoid direct beam contribution, the aperture was shifted laterally by 60 *μ*m.

The overfocused direct beam measurement was performed with a high-resolution camera (pixel size of about 1.8 *μ*m) located at 1.72 m from the lens focal plane. The intensity pattern is the result of 100 acquisitions of 1s each, where the zero photon background has been subtracted. The inversion of the intensity data is performed as described in Ref. 29, using a rough estimate of the beam profile as a starting guess. Numerical tests introducing photon shot noise show that a solution of relevant quality is obtained even if the support knowledge is known within ±15%.

### Experiment: 3D Bragg ptychography acquisition

The sample was mounted vertically onto a three-axis piezo stage, fixed on the top of an hexapod. The accurate alignment of the sample center of rotation with regards to the focal plane was ensured by the use of an optical microscope with a depth of focus of about 1 *μ*m. The sample was rotated to the Bragg angle 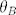 of the 220 reflection (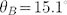), which resulted in an elongated focal spot size of 1.2 × 0.23 *μ*m^2^, as schematically shown in Figure 1b.

The intensity acquisition was performed with a Maxipix single photon detector (516 x 516 pixels of 55 *μ*m size), located at 2.25 m from the sample. At each position of the beam onto the sample, the sample was rotated in steps of 0.013°, along the rocking curve. This angular sampling allowed to ensure that the exit field was fully contained into the conjugated space associated to the detector frame^21^. The 20 frames were then stacked in order to provide the full 3D intensity pattern, for a given beam to sample position. Each intensity pixel was associated to a reciprocal space vector 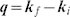, with coordinates 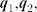 and 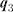. Precisely, 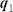 and 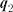 are defined along the detector frame, parallel and perpendicular to the incident plane, respectively, while 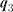 corresponds to the direction of the rocking curve, *i.e.*, is tangent to the 220 Bragg vector.

In order to acquire partially redundant information, the sample position 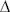 was scanned along *y* in steps of 50 nm (Figure 1b), resulting in an overlapping of 78 %. The total scanning range (21 steps) ensured that the whole structure was explored. The acquisition time (30 s per frame) led to an intensity maximum of about 5700 photons/pixel.

### Inversion of the experimental data set

The inversion of the intensity data set was performed directly in the detector frame in order to keep the truly measured intensity value in each detector pixel. It preserves thereby the statistical properties of the photon shot-noise corrupted signal. Consequently, the conjugated direct space frame into which all the parameters linked to the sample are described is non-orthogonal^21^. During the inversion procedure, the illumination function was kept fixed. Its 3D distribution was derived from the beam profile estimated in Figure 2, which was considered as constant along the propagation depth into the sample.

The inversion cycle was initialized by the sample starting estimate, a strain-free structure with external shape close to the SOI pattern nominal shape. In addition, in order to avoid the reconstruction of ambiguous solutions due to the lack of diversity along the beam propagation direction, the search of regularized solutions was enforced^21^, penalizing the reconstruction of the object outside a film-like support (0.2 *μ*m thick). The conjugate gradient algorithm was used for the inversion, together with a *Bouman and Sauer* description of the shot-noise probability distribution function^34^,^33^. This latter was chosen for its capacity to emphasize the low frequency components, leading to smooth solutions. Other noise models were tested, resulting qualitatively to the same results.

### Transmission electron microscopy (TEM)

High-resolution TEM observations were obtained on a JEOL 2200FS microscope equipped with an ultra-high resolution pole piece and working at 200 keV. The 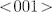 cross-sectional preparations have been done by mechanical polishing followed by an argon ion beam milling until the electron transparency (with a low voltage of 2 kV at the end, to limit the thickness of amorphous layer on the sides of the preparation). Strain mapping analysis were performed using the geometrical phase analysis (GPA) software (for more informations see Refs. [35, 36]). The z axis of the sample (Figure 1) corresponds to the 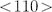 direction.

## Author Contributions

The research project was designed by VC. The sample was prepared by AT and the TEM experiments were performed by GP. MB prepared the experimental set-up at the synchtrotron and performed the x-ray measurements together with VC. VC analysed the data together with the help of MA. The manuscript was written by VC, PG and MA with the help of all others.

## Additional Information

**How to cite this article**: Chamard, V. *et al*.Strain in a silicon-on-insulator nanostructure revealed by 3D x-ray Bragg ptychography. *Sci. Rep.* **5**, 9827; doi: 10.1038/srep09827 (2015).
